# Sulforaphane Protect Against Cadmium-Induced Oxidative Damage in mouse Leydigs Cells by Activating Nrf2/ARE Signaling Pathway

**DOI:** 10.3390/ijms20030630

**Published:** 2019-02-01

**Authors:** Shu-Hua Yang, Peng Li, Li-Hui Yu, Lin Li, Miao Long, Ming-Da Liu, Jian-Bin He

**Affiliations:** 1 Key Laboratory of Zoonosis of Liaoning Province, College of Animal Science & Veterinary Medicine, Shenyang Agricultural University, Shenyang 110866, China; yangshuhua0001@syau.edu.cn (S.-H.Y.); lipeng2018@syau.edu.cn (P.L.); yuyang75060@163.com (L.-H.Y.); lilin619619@163.com (L.L.); 2 College of Land and Environmental Sciences, Shenyang Agricultural University, Shenyang 110866, China

**Keywords:** cadmium, Nrf2/ARE signaling pathway, oxidative damage, SFN, TM3 cell

## Abstract

Cadmium (Cd) is harmful for humans and animals, especially for the reproductive system. However, the mechanism of its toxicity has not been elucidated, and how to alleviate its toxicity is very important. This study aimed to explore the role and mechanism of action of sulforaphane (SFN) in protecting mouse Leydigs (TM3) cells from cadmium (Cd)-induced damage. The half-maximal inhibitory concentration (IC_50_) of Cd and the safe doses of SFN were determined using a methyl thiazolyl tetrazolium (MTT) assay. The testosterone secretion from TM3 cells was measured using the enzyme-linked immunosorbent assay. The intracellular oxidative stress was evaluated using corresponding kits. The cell apoptosis was detected using flow cytometry. The mRNA expression of genes associated with NF-E2-related factor 2 (Nrf2)/antioxidant response element (ARE) signaling was detected using reverse transcription–polymerase chain reaction, including Nrf2, heme oxygenase I (HO-1), glutathione peroxidase (GSH-Px), NAD(P)H:quinone acceptor oxidoreductase 1 (NQO1), and γ-glutamylcysteine synthetase (γ-GCS). The protein expression of Nrf2, GSH-Px, HO-1, γ-GCS, and NQO1 was detected using Western blot analysis. The results showed that the IC_50_ of Cd to TM3 cells was 51.4 µmol/L. SFN reduced the release of lactate dehydrogenase from Cd-exposed cells. Cd + SFN 2.5 treatment significantly elevated testosterone concentration compared with the Cd group (*p* < 0.05). SFN significantly increased total superoxide dismutase (T-SOD) and GSH-Px activity and GSH content in Cd-treated cells (*p* < 0.05; *p* < 0.01), inhibited the production of malondialdehyde or reactive oxygen species caused by Cd (*p* < 0.05; *p* < 0.01), and reduced the apoptotic rate of Cd-induced TM3 cells (*p* < 0.01). SFN upregulated the mRNA expression of *Nrf2*, *GSH-Px*, *HO-1*, *NQO1*, and *γ-GCS* in Cd-treated cells, indicating the protective effect of SFN against Cd-induced oxidative stress or cell apoptosis by activating the Nrf2/ARE signaling pathway.

## 1. Introduction

Cadmium (Cd) is a toxic heavy metal often found in industrial and agricultural pollutants. It could be absorbed by animals from contaminated food, water and air. The accumulation of Cd causes damage to the body [1,2]. Cd is toxic to the kidney, liver, bone, lung, and testis. Damage to testis is the major harm of chronic Cd accumulation [3,4,5]. The reproductive toxicity of Cd manifests mainly as reduced testis weight, atrophy of seminiferous tubules, impaired sperm production, decreased sperm motility, increased sperm deformity, and lowered testosterone secretion [6,7,8,9,10]. Cd can also affect sperm fertilization and embryo development [11,12].

Sulforaphane (SFN) is an organic sulfur compound derived from crucifers such as broccoli, cabbages and olives. It is one of the naturally active substances with the best anticancer effect [13,14,15]. It is also one of the antioxidants with the highest efficiency [16,17] because it significantly reduces tissue or structural damages caused by oxidative stress [18]. SFN could activate a series of phase II enzymes and antioxidant enzymes that could react with active oxygen molecules. Compared with small-molecule antioxidants, such as vitamin C or K, phase II enzymes and antioxidant enzymes could persist for a longer time. Thus, the antioxidant effect of SFN persisted long after absorption [19]. Previous studies confirmed that SFN can antagonize Cd-caused kidney damage by inducing the synthesis of antioxidant enzymes [20], improve hepatocyte activity by reducing the accumulation of arsenic in liver tissues [21], and prevent diabetes-caused reproductive disorders by upregulating the expression of Nrf_2_ in testicular cells [22]. An in vivo study also found that SFN reduced testis damage and improved spermatogenic function in mice with Cd poisoning [23], but its underlying mechanism is yet to be understood.

Considering the antioxidant properties of SFN, mouse TM3 cells were used in this study to analyze the effects and mechanism of action of SFN in antagonizing Cd-induced cell damage, thereby providing the theoretical basis for developing a safe and reliable way for controlling and preventing Cd-induced reproductive toxicity.

## 2. Results

### 2.1. The Survival Rate of Cd-Induced TM3 Cells

**Figure 1:**
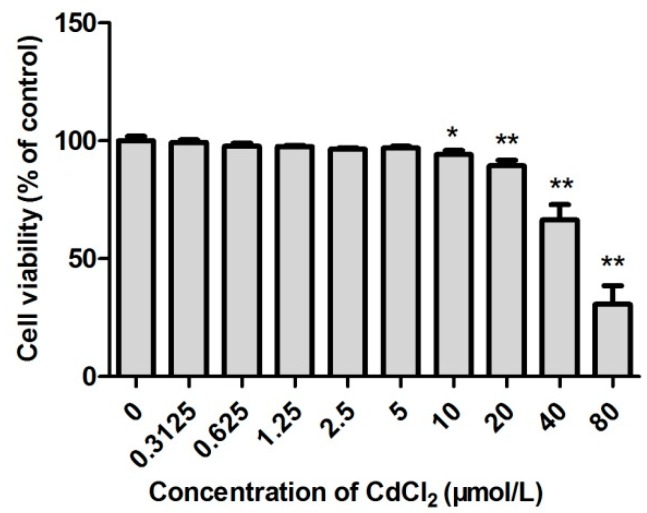
Effect of cadmium on the survival rate of TM3 cells. * and ** represent a significant and an extremely significant difference, respectively, compared with the control group.

As shown in Figure 1, the survival rate of cells treated with 0.3125, 0.625, 1.25, 2.5, and 5 µmol/L Cd was insignificantly different from that of the control group (*p* > 0.05). However, 10 µmol/L Cd significantly reduced the survival rate of cells (*p* < 0.05), and 20 µmol/L Cd resulted in an extremely low survival rate of TM3 cells (*p* < 0.01). Based on the results, the IC_50_ of Cd for TM3 cells was determined as 51.4 µmol/L.

### 2.2. Survival Rate of SFN-Treated TM3 Cells

**Figure 2:**
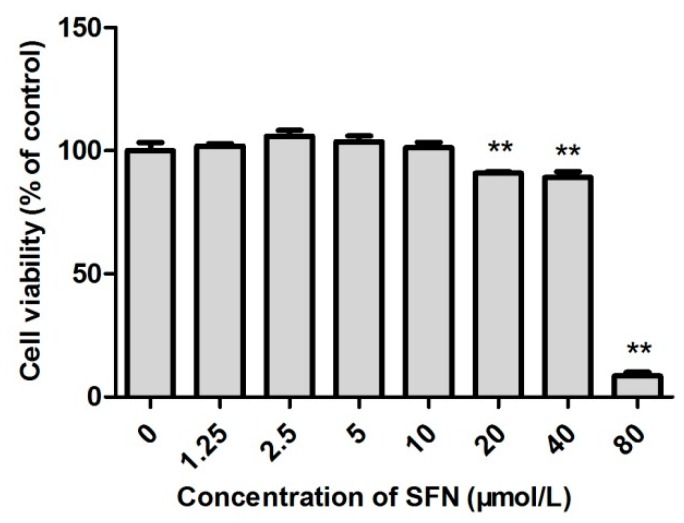
Effect of sulforaphane on the survival rate of TM3 cells. * and ** represent a significant and an extremely significant difference, respectively, compared with the control group.

As shown in Figure 2, the survival rate of cells treated with 1.25, 2.5, 5, and 10 µmol/L SFN was higher than that of the control group. The survival rate peaked 105.9% when the SFN concentration was 2.5 µmol/L. However, 20, 40 and 80 µmol/L SFN significantly reduced the cell survival (*p* < 0.01).

### 2.3. Survival Rate of TM3 Cells Treated with Cd + SFN

**Figure 3:**
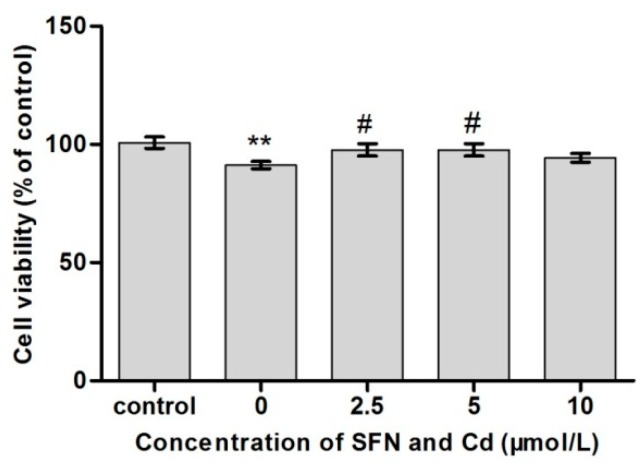
Influence of Cd and SFN on the survival of TM3 cells. The cells were treated with 0, 2.5, 5, and 10 µmol/L SFN and 10 µmol/L Cd. ** represent an extremely significant difference, compared with the control group. ^#^ represents a significant difference, compared with the Cd group.

As shown in Figure 3, cell survival was significantly lower in the Cd group (*p* < 0.01). Although the cells in the Cd + SFN2.5(and 5.0) groups had a decreased survival rate compared with the control group (*p* < 0.05), they still had higher rates of survival compared with the Cd treatment groups.

### 2.4. LDH Activity in TM3 Cells Treated with Cd + SFN

**Figure 4:**
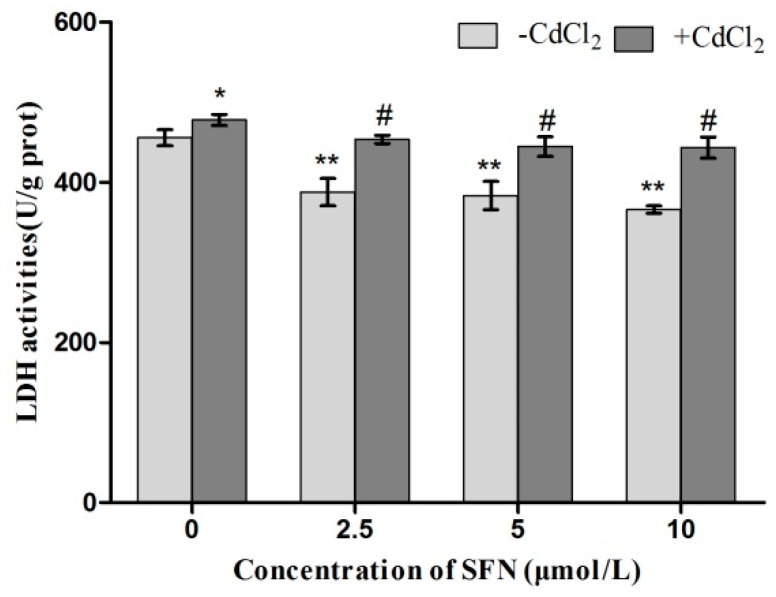
Activity of LDH in TM3 cells. * and ** represent a significant and extremely significant difference, respectively, compared with the control group. ^#^ represents a significant difference, compared with the Cd group.

As shown in Figure 4, the LDH activity was significantly higher in the Cd group (*p* < 0.05) and lower in SFN groups (*p* < 0.01) compared with the control group. Besides, it decreased in a dose-dependent manner with the increase in the SFN concentration. The LDH activity was significantly lower in the Cd + SFN groups than in the Cd group (*p* < 0.05).

### 2.5. Concentration of Testosterone

**Figure 5:**
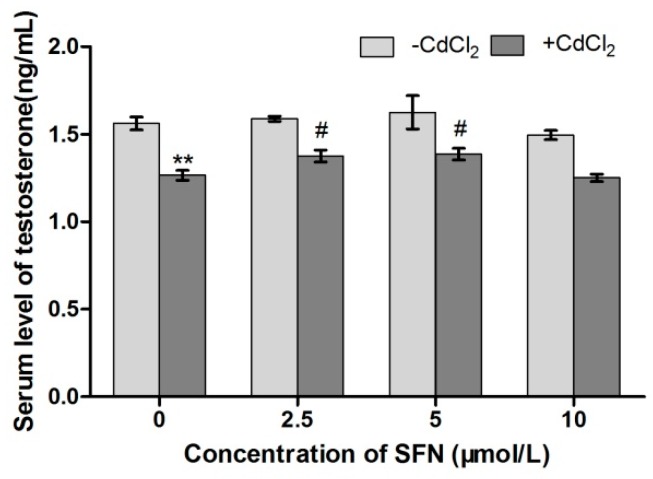
Secretion levels of testosterone in TM3 cells. * and ** represent a significant and extremely significant difference, respectively, compared with the control group. ^#^ represents a significant difference, compared with the Cd group.

The effects of Cd and SFN on testosterone secretion in TM3 cells were measured using ELISA. Compared with the control group, the concentration of testosterone significantly decreased in Cd-treated cells (*p* < 0.01) and insignificantly increased in SFN-treated cells (*p* > 0.05). Compared with the Cd group, the level of testosterone was significantly higher in the Cd + SFN2.5 and Cd + SFN5 groups (*p* < 0.05). Also, the level in the Cd + SFN10 groups was insignificantly different from that in the Cd group (*p* > 0.05) (Figure 5).

### 2.6. TM3 Cell Apoptosis

**Figure 6:**
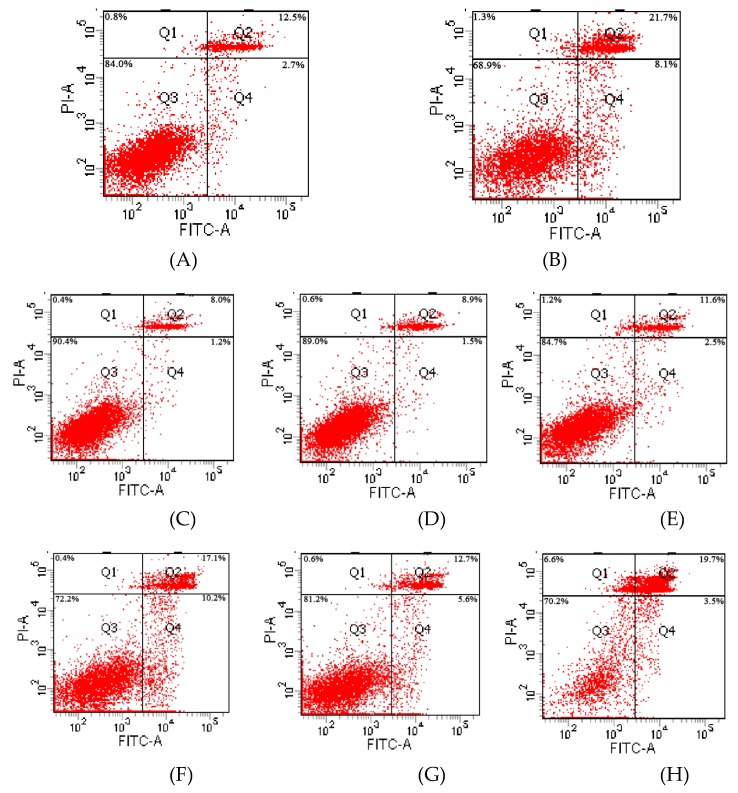
Scatter plot of cell cytometry. (**A**) Control group; (**B**) Cd group; (**C**) SFN2.5 group; (**D**) SFN5 group; (**E**) SFN10 group; (**F**) Cd + SFN2.5 group; (**G**) Cd + SFN5 group; and (**H**) Cd + SFN10 group.

**Figure 7:**
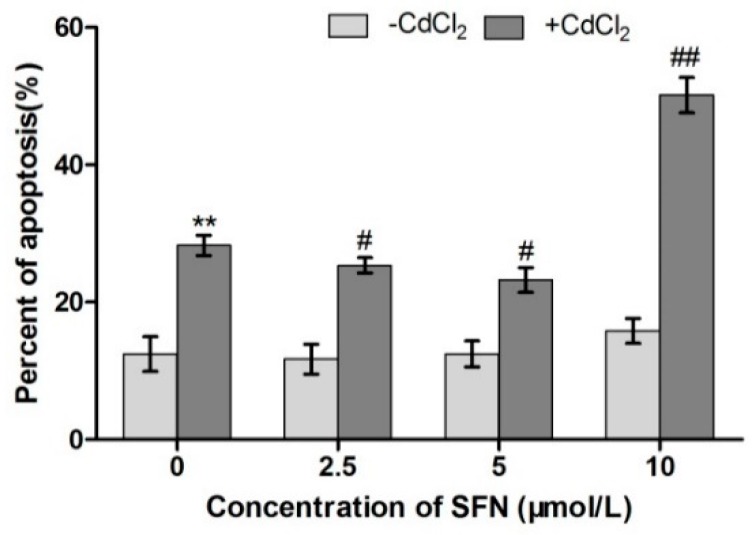
Detection of cell apoptosis using FITC Annexin V/PI double-labeling (%). ** represents an extremely significant difference, compared with the control group. ^#^ and ^##^ represent a significant and an extremely significant difference, respectively, compared with the Cd group.

Flow cytometry with FITC Annexin V/PI double-labeling was performed to evaluate the influence of Cd and SFN on TM3 cell apoptosis. Figure 6 and Figure 7 show the results of cell apoptosis and data analysis, respectively.

Q2 represents cells in late apoptosis, and Q4 represents cells in early apoptosis. The greater density of the red scatter points indicates a higher degrees of cell apoptosis. The density of red scatter points in Q2 and Q4 was significantly higher in the Cd group than in the other groups. The Cd + SFN groups had more scatter points compared with the control group. The SFN groups had the least scatter points. As shown in Figure 7, the apoptosis rate was significantly higher in the Cd group than in the control group (*p* < 0.01), whereas 2.5 and 5 µmol/L SFN resulted in a level of apoptosis lower than that in the control group (*p* > 0.05). Compared with the Cd group, the apoptosis of TM3 cells in the Cd + SFN2.5, Cd + SFN5 and Cd + SFN10 groups decreased, and the difference was extremely significant for the first two groups (*p* < 0.05) and significant for the last group (*p* < 0.01).

### 2.7. Detection of ROS Released by TM3 Cells

**Figure 8:**
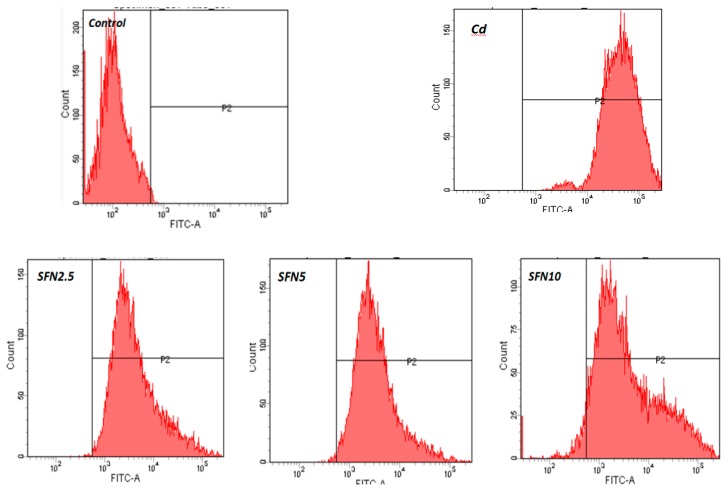

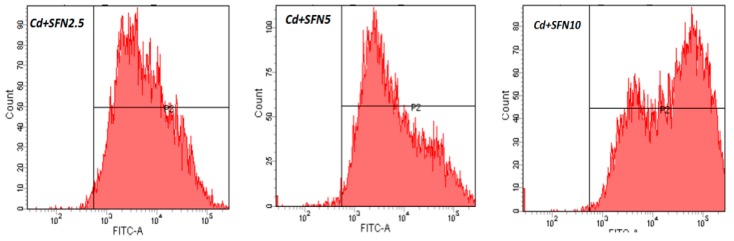
Level of ROS in TM3 cells.

**Figure 9:**
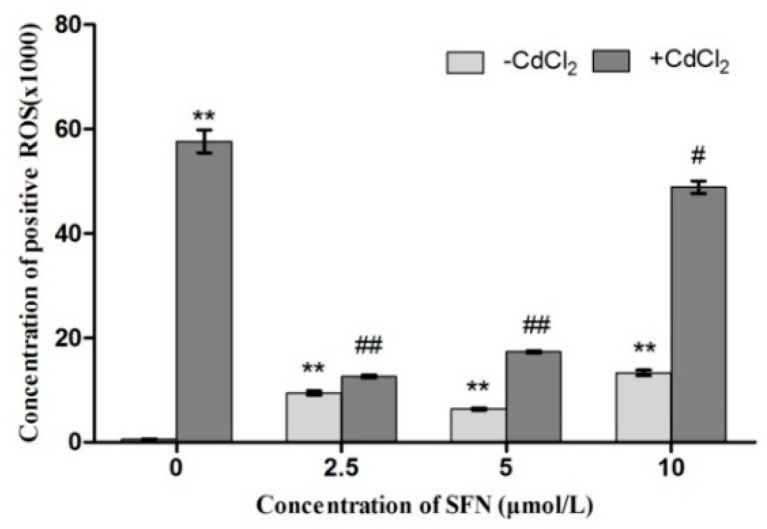
Detection of ROS using a DCFH-DA fluorescent probe. ** represents an extremely significant difference, compared with the control group. ^#^ and ^##^ represent a significant and an extremely significant difference, respectively, compared with the Cd group.

ROS were detected using flow cytometry. P2 represented positive ROS release from TM3 cells (Figure 8). As shown in Figure 8, the control showed an extremely low level of ROS release, with almost all cells distributed in the negative area. The Cd group had the highest positive rate, with cells distributed on the right side of the P2 area; other groups also showed varying degrees of ROS increase (Figure 8). The results of data analysis are shown in Figure 9. Compared with the control group, all experimental groups had an increased ROS release (*p* < 0.01), and the difference was most significant in the Cd group (*p* < 0.01). Compared with the Cd group, the levels of ROS release in the SFN and Cd + SFN groups were significantly lower (*p* < 0.01 or *p* < 0.05) (Figure 9).

### 2.8. Detection of Cell Antioxidant Ability

**Figure 10:**
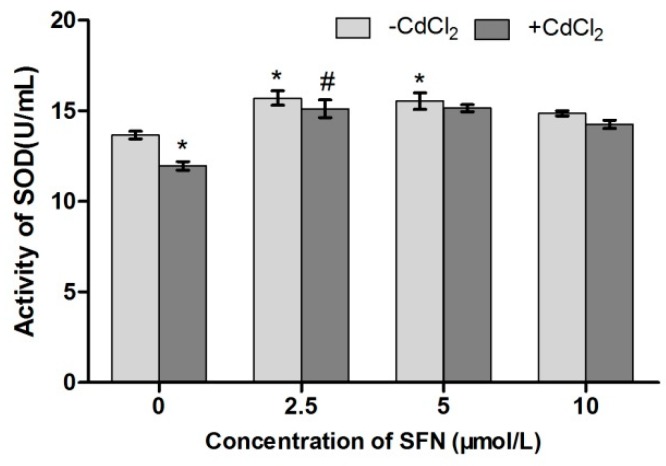
Activity of SOD in TM3. * represent a significant difference, respectively, compared with the control group. ^#^ represent a significant difference compared with the Cd group.

**Figure 11:**
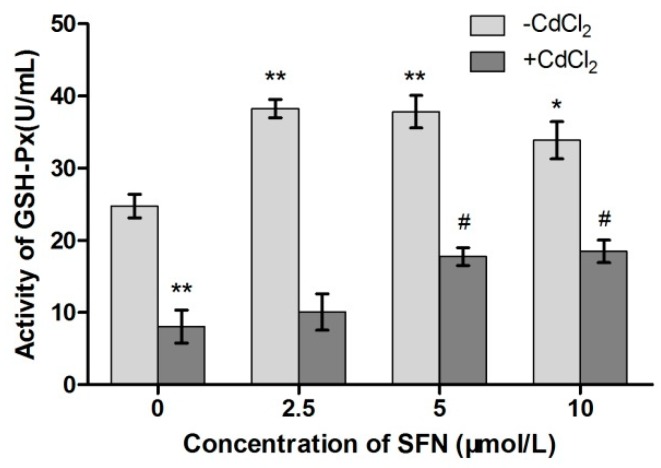
Activity of GSH-PX in TM3. * and ** represent a significant and an extremely significant difference, respectively, compared with the control group. ^#^ represent a significant difference compared with the Cd group.

**Figure 12:**
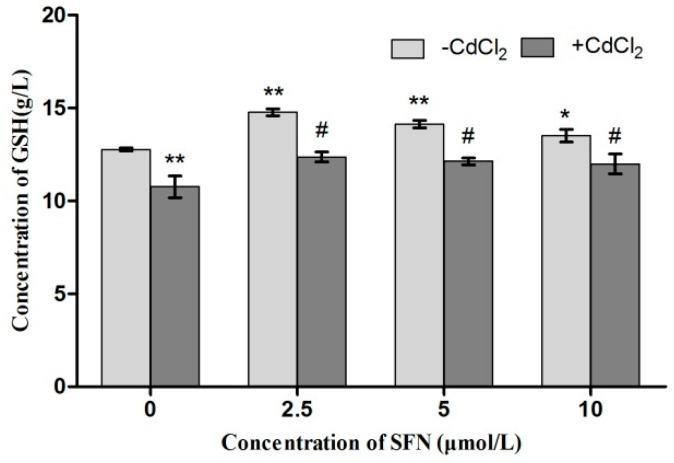
Content of GSH in TM3. * and ** represent a significant and an extremely significant difference, respectively, compared with the control group. ^#^ represent a significant difference compared with the Cd group.

**Figure 13:**
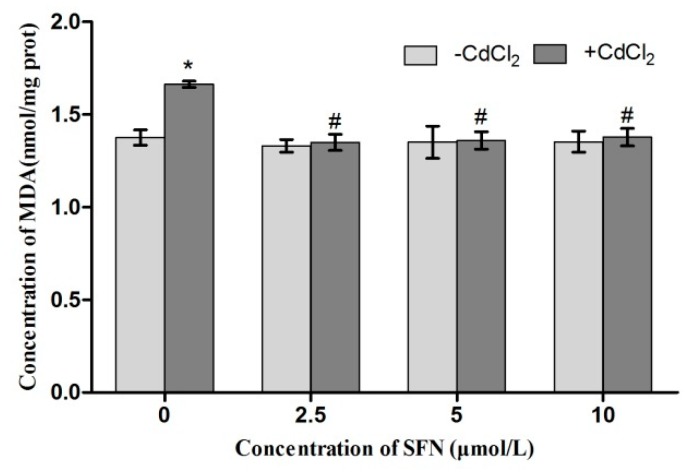
Content of MDA in TM3. * represent a significant difference, respectively, compared with the control group. ^#^ represent a significant difference compared with the Cd group.

Compared with the control group, the activity of SOD and GSH-Px in Cd-treated cells was significantly lower (*p* < 0.05, *p* < 0.01). The SOD activity in SFN-treated cells showed a rising trend (*p* < 0.05), and the activity of GSH-Px significantly increased (*p* < 0.01, *p* < 0.05). Compared with the Cd group, the activity of SOD in the Cd + SFN2.5 group showed a rising trend (*p* < 0.05). The GSH-Px activity was significantly higher at SFN concentrations of 5 and 10 µmol/L (*p* < 0.05) (Figure 10 and Figure 11). Compared with the control group, the GSH content was significantly lower in the Cd group (*p* < 0.01) and significantly different in the SFN 2.5, SFN 5, and SFN 10 groups, with *p* < 0.01 for the first two groups and *p* < 0.05 for the last. The GSH level was slightly higher in the Cd + SFN group than in the Cd group (*p* < 0.05) (Figure 12). After Cd induction, the content of MDA increased in TM3 cells (*p* < 0.05). The addition of SFN reduced the level of MDA, with a significant difference identified in the Cd + SFN groups (*p* < 0.05) (Figure 13).

### 2.9. Expression of Genes Involved in the Nrf2 Pathway

**Figure 14:**
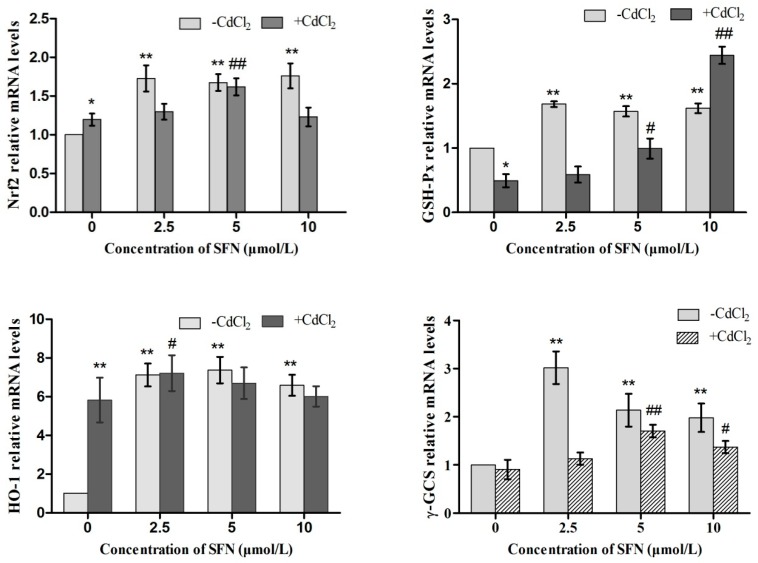

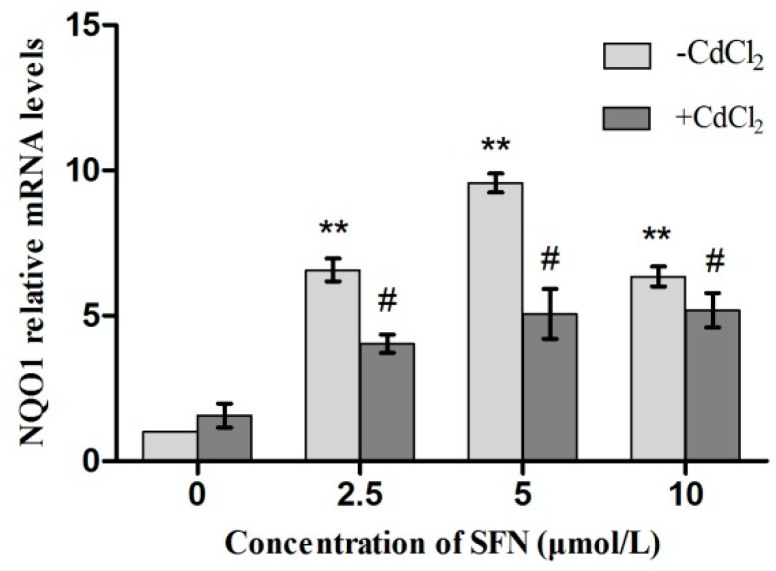
Effects of SFN on Cd induced the relative mRNA expression of *Nrf2*, *GSH-Px*, *HO-1*, *γ-GCS*, and *NQO1* of TM3 cells. * and ** represent a significant and an extremely significant difference, respectively, compared with the control group. ^#^ and ^##^ represent a significant and an extremely significant difference, respectively, compared with the Cd group.

Short-term Cd and SFN treatment could induce the mRNA expression of *Nrf2*, *HO-1*, *γ-GCS*, *GSH-Px*, and *NQO1*. Compared with the control group, SFN-treated cells had upregulated expressions of the aforementioned genes, with *Nrf2*, *HO-1*, *γ-GCS*, *GSH-Px*, and *NQO1* showing significant differences (*p* < 0.01). Cd-treated cells also showed increased expression, except for *GSH-Px* and γ-GCS. The expression of *Nrf2*, *HO-1*, *GSH-Px*, *γ-GCS*, and *NQO1* was higher in the Cd+SFN group than in the Cd group. Compared with the Cd group, there was a significant difference in the expression of *GSH-Px* and *γ-GCS* in the Cd+SFN5 (and 10) groups (*p* < 0.05, *p* < 0.01), the expression of *Nrf2* in the Cd+SFN5 group (*p* < 0.01), the expression of *HO-1* in the Cd+SFN2.5 group (*p* < 0.05), and the expression of *NQO1* in the Cd+SFN group (*p* < 0.01) (Figure 14).

### 2.10. Expression of Proteins Involved in the Nrf2 Pathway

**Figure 15:**
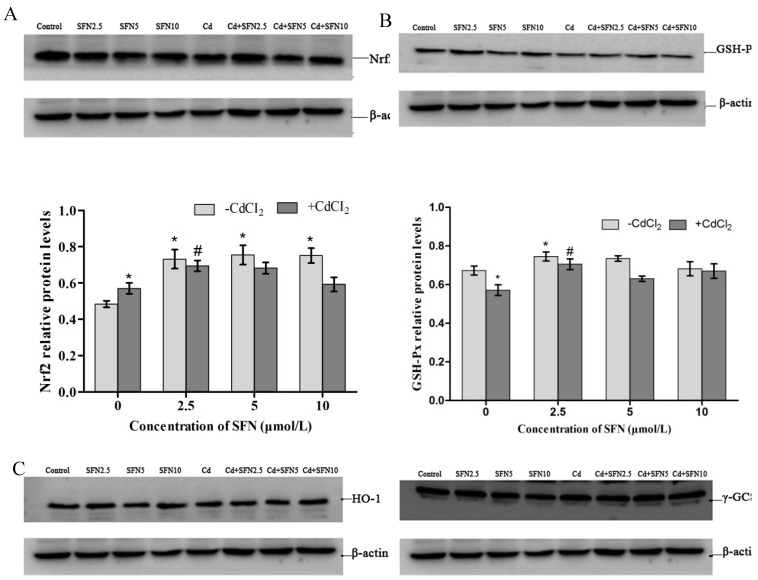

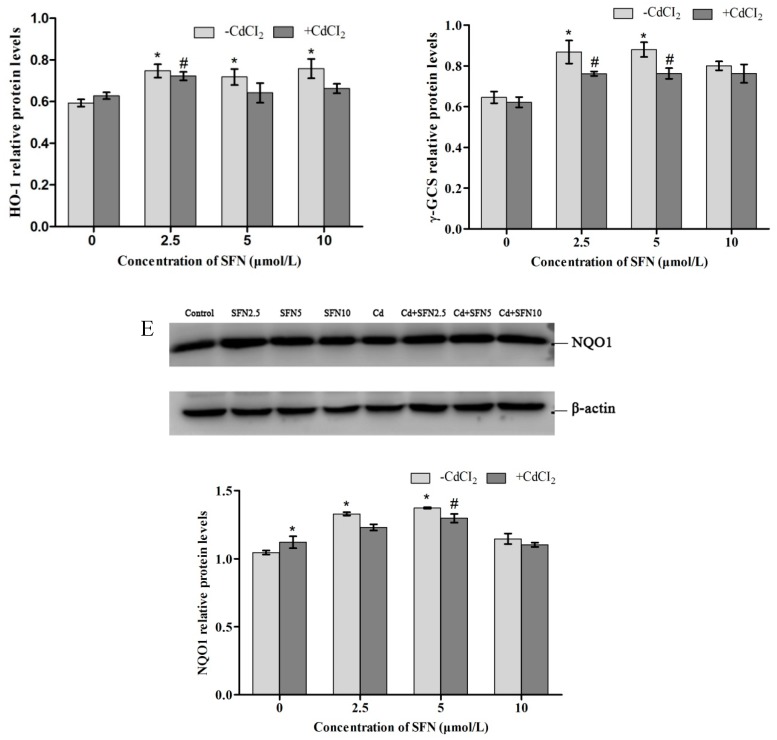
Effects of SFN on Cd induced the protein expression of (**A**) Nrf2, (**B**) GSH-Px, (**C**) HO-1, (**D**) γ-GCS, and (**E**) NQO1 of the TM3 cells. * represents a significant difference compared with the control group. ^#^ represents a significant difference compared with the Cd group.

The protein expression of Nrf2, GSH-Px, HO-1, γ-GCS, and NQO1 was consistent with mRNA expression. Compared with the control group, SFN-treated cells had an increased expression of all proteins (*p* < 0.05, *p* > 0.05). Cd-treated cells also had upregulated protein expression, except for GSH-Px andγ-GCS. The Cd + SFN groups had higher levels of Nrf2, GSH-Px, HO-1, γ-GCS, and NQO1, but the difference was not significant (*p* > 0.05) (Figure 15).

## 3. Discussion

Cd is an important environmental pollutant that has a significant influence on the health of humans and livestock [24,25,26]. In particular, Cd can damage the liver, kidney and vascular system. Recent studies showed that Cd exposure significantly impaired the structure and function of reproductive organs in male animals [27,28,29]. SFN is an isothiocyanic acid mainly presenting in broccoli and other cruciferous vegetables. It has anti-tumor, anti-apoptotic, anti-oxidative, anti-inflammatory, and anti-bacterial effects [17,30,31,32]. In this study, TM3 cells were used to analyze the effect and mechanism of action of SFN in antagonizing Cd-induced cytotoxicity via its antioxidant properties. The safe concentrations for Cd and SFN were determined by measuring the relative survival rate of TM3 cells. The IC_50_ of Cd for TM3 cells was 51.4 µmol/L. Therefore, 1/5 IC_50_ (10 µmol/L) was used as the concentration for induction. Also, 0–10 µmol/L SFN elevated the survival rate of TM3 cells. Yet, SFN at a concentration of more than 20 µmol/L negatively impacted the survival of cells, indicating that surpassing a certain concentration, SFN might cause damage to cells. TM3 cells were cultured in Cd (10 µmol/L) and SFN (2.5, 5, and 10 µmol/L) to determine whether SFN improved Cd-induced cytotoxicity, yielding a survival rate significantly higher than that in the Cd group. Thus, the experimental model with co-culturing in Cd and SFN was established.

Previous studies showed that Cd induced endocrine dysfunction in animals, and exposure to Cd significantly reduced the level of testosterone secretion [23,33]. The in vitro culture of TM3 cells also provided a consistent result; Cd-treated TM3 cells had a significantly lowered testosterone concentration compared with the control group. Interestingly, the level of testosterone in cells treated with SFN and Cd + SFN showed varying degrees of elevation compared with those treated with Cd, indicating that SFN, in some way, improved the survival of TM3 cells and promoted the synthesis and secretion of testosterone. Based on this hypothesis, a series of experiments were carried out.

LDH is an intracellular enzyme released when cells rupture or are damaged. Thus, the degree of cell damage can be evaluated by detecting the LDH activity in the culture medium [34]. TM3 cells cultured with Cd alone had significantly increased LDH activity (*p* < 0.05), whereas the addition of SFN significantly lowered LDH activity in the supernatant (*p* < 0.01). The LDH activity was significantly reduced in the Cd + SFN groups than in the Cd group (*p* < 0.05, *p* < 0.01) (Figure 4), suggesting that SFN inhibited Cd-induced damage to the cell membrane and reduced the release of LDH from TM3 cells.

Previous studies reported that treating human peripheral blood lymphocytes and monocytes with Cd and SFN improves Cd-induced cell apoptosis and necrosis [35]. A similar result was obtained in studying human mesenchymal stem cells [36]. The present study used flow cytometry with Annexin V–FITC and PI double-labeling to evaluate cell apoptosis. It found that Cd induced cell apoptosis, whereas SFN decreased the apoptosis rate of Cd-treated TM3 cells. When the concentration of SFN was 2.5 and 5 µmol/L, the apoptotic rate of TM3 cells was significantly lower compared with that in the Cd group (*p* < 0.05) (Figure 7), indicating that SFN reduced Cd-induced cytotoxicity in the in vitro culture. Also, Cd-treated cells decreased the ability to secrete testosterone, which might be attributed to the increased degree of cell apoptosis.

Previous studies suggested that Cd could induce oxidative stress to many tissues or organs, thereby affecting certain physiological functions. For example, Cd caused oxidative stress to the olfactory system of zebrafish [37] and to the kidney [4,20], spleen [38], and testis of rats [7,23,39,40]. In this study, Cd disrupted the oxidation–reduction equilibrium in TM3 cells, reduced SOD and GSH-Px activity and GSH content, and promoted the synthesis of lipid peroxidation products, namely MDA (Figure 10, Figure 11, Figure 12 and Figure 13). Flow cytometry was performed to detect ROS release from TM3 cells so as to explore the mechanism by which Cd induced oxidative stress. The results showed that cells treated with Cd had significantly increased ROS release, indicating that Cd impaired the antioxidant defense system of cells by elevating the level of ROS and further caused oxidative stress and subsequent cellular damage (Figure 4) or apoptosis (Figure 7). The present study found that cells cultured with SFN or Cd + SFN had a significantly lowered degree of ROS release (*p* < 0.01 or *p* < 0.05) (Figure 9), with increased SOD and GSH-Px activity and GSH content and reduced lipid peroxidation damage (Figure 10, Figure 11, Figure 12 and Figure 13) or cell apoptosis (Figure 7). This might be because Cd can bind to the sulfhydryl on GSH, SOD, or GSH-Px, thus inhibiting the clearance of lipid peroxides and inducing oxidative stress. This also suggested a significant role of SFN in preventing Cd-induced oxidative stress, but the specific mechanisms need further investigation.

Nrf2–ARE is one of the most important antioxidant pathways [41,42], where Nrf2 functions as a key factor in initiating Nrf2–ARE signaling [43]. The expression of important genes involved in this pathway was analyzed to understand whether SFN antagonized Cd-induced cytotoxicity by activating the Nrf2–ARE pathway. The results showed that the cells independently treated with Cd or SFN demonstrated an increasing trend in the expression of Nrf2-signaling genes, with varying degrees of upregulation identified in either mRNAs or proteins of HO-1, NQO1 and γ-GCS. The mRNA and protein levels of Nrf2, HO-1, NQO1, and γ-GCS were higher in the Cd + SFN groups than in the Cd group (Figure 14 and Figure 15). The results suggested that in vitro short-term stimulation by Cd and SFN could activate the Nrf2–ARE signaling pathway and elevate the expression of downstream mRNAs and proteins, thereby inhibiting oxidative stress and preventing cell damage. The results of the present study were consistent with the findings of Yang et al. (2016) [23], who showed that SFN significantly upregulated the levels of Nrf2, HO-1 and NQO1 and reduced the apoptosis of testicular cells. However, the expression of GSH-Px mRNA and protein showed an opposite trend, with significantly lowered levels in Cd-treated cells. Also, the expression of GSH-Px was higher in the Cd + SFN groups than in the Cd group. It was speculated that Cd, either by competitive or noncompetitive substitution, could replace the active cofactors (metal ions) for intracellular metal-dependent enzymes, such as Se in GSH-Px, leading to the decreased expression of GSH-Px. Since Cd has a high affinity for the sulfhydryl group, it could bind with selenocysteine on GSH-Px, thereby reducing the protein level of GSH-PxThus, the expression of GSH-Px was lower in Cd-treated TM3 cells than in the control group in the present study.

In conclusion, this study showed that Cd could promote oxidative damage and apoptosis in TM3 cells, whereas SFN could attenuate Cd-induced TM3 cell damage by activating the Nrf2/ARE signaling pathway. SFN changed the ability of TM3 cells to secrete testosterone and directly affected the spermatogenesis of mice. The results of this study provide a reference for the application of SFN in preventing and treating Cd-induced reproductive toxicity in males.

## 4. Materials and Methods

### 4.1. Treatment of TM3 Cells with Cd and SFN

TM3 cells (purchased from ATCC, Manassas, Virginia, USA) were seeded in 10% FBS-containing DMEM/F12 medium (purchased from HyClone, Logan City, Utah, USA) and cultured at 37 °C with 5% CO_2_ for 24 h. The cell suspension (2 × 10^5^/mL) was inoculated into a 96-well plate and cultured for 24 h. Then, the cells were moved to a medium containing 0, 0.3125, 0.625, 1.25, 5, 10, 20, 40, and 80 µmol/L of Cd to determine the half-maximal inhibitory concentration (IC_50_). They were cultured with 0, 1.25, 5, 10, 20, 40, and 80 µmol/L SFN (LKT Inc, St. Paul, MN, USA) for 24 h to measure the relative survival rate and determine the safe concentration of SFN for TM3 cells.

The cells were adjusted to a concentration of 2 × 10^5^/mL and inoculated in a 96-well plate for independent or combined culturing with Cd at 1/5 IC_50_ (10 µmol/L Cd) and SFN at three safe concentrations (2.5, 5.0 and 10.0 µmol/L) to determine the relative survival rate of TM3 cells.

The cells were adjusted to a concentration of 1 × 10^6^ /mL and inoculated in a 6-well plate with Cd and SFN to determine TM3 cell activity, testosterone secretion, antioxidant ability, cell apoptosis, and the expression of genes involved in the Nrf2/ARE signaling pathway. Each experiment was repeated four times.

### 4.2. Determining the Relative Survival Rate of TM3 Cells

The cells were inoculated in a 96-well plate, grouped, and cultured for 24 h with Cd and SFN independently or in combination. The relative survival rate was determined using the MTT Cell Proliferation and Cytotoxicity Assay Kit according to the manufacturer’s protocol (Beijing solarbio science & technology co ltd, Beijing, China). The absorbance at 570 nm was measured using a plate reader.

### 4.3. Determining LDH Activity

The cells were inoculated in a 6-well plate, grouped, and cultured for 24 h. The supernatant was used to detect LDH activity according to the instructions provided with the 2,4-dinitrophenylhydrazine colorimetric kit (Nanjing Jiancheng Biotechnology Institute, Nanjing, China).

### 4.4. Measuring Testosterone Concentration

The effect of Cd and SFN on testosterone secretion was measured using the enzyme-linked immunosorbent assay (ELISA) according to the instructions provided with the testosterone (T) ELISA kit (Wuhan Elabscience Biotechnology Co ltd, Wuhan, China).

### 4.5. Detecting Antioxidant Ability

The cells were grouped and cultured for 24 h. The supernatant was used for detecting lipid peroxidation indexes. The SOD activity was detected using xanthine oxidase. GSH-Px activity and GSH content were detected using 5,5′-dithiobis (2-nitrobenzoic acid). The MDA content was detected using thiobarbituric acid. Kits were obtained from the Nanjing Jiancheng Institute of Biotechnology (Nanjing, China). Reactive oxygen species (ROS) were detected using a Dichloro-dihydro-fluorescein diacetate (DCFH-DA) fluorescent probe (Beijing solarbio science & technology co ltd, Beijing, China). All experiments were performed according to the manufacturers’ protocols.

### 4.6. Quantification of Cell Apoptosis

Cell apoptosis was quantified by flow cytometry using the Annexin V–FITC/propidium iodide (PI) double-labeling method (Dakewe Biotech Co ltd, Beijing, China). The cells were grouped and cultured. Each group had four repeats with another three controls for correction. After culturing, the cells were digested using EDTA-free trypsin, washed twice with 0.1M PBS, resuspended in 300 µL of Annexin V binding buffer, mixed, and incubated with 5 µL of FITC Annexin V and 10 µL of PI solution for 15 min in the dark at room temperature. The fluorescent emission from FITC and PI was detected at 530 and 585 nm, respectively. Q1: (Annexin V–FITC)–/PI+, cells were necrotic; Q2: (Annexin V + FITC)+/PI+, cells were in late apoptosis; Q3: (Annexin V–FITC)−/PI−, cells were alive; Q4: (Annexin V–FITC)+/PI−, cells were in early apoptosis. Therefore, the total apoptotic proportion included the percentage of cells with fluorescence (Annexin V–FITC)+/PI− and (Annexin V + FITC)+/PI+.

### 4.7. Expression of Genes Involved in the Nrf2/ARE Signaling Pathway

Total RNA was extracted from TM3 cells using the RNA extraction kit (Sangon, Shanghai, China). Real-time PCR was performed to detect mRNA expression of Nrf2, GSH-Px, NQO1, γ-GCS, and HO-1 in TM3 cells. RNA was purified using the OD260/280 ratio. Real-time RT-PCR data was calculated using the following gene expression formula 2^−ΔΔ*C*t^. All values were normalized using β-actin as a reference. Real-time PCR was conducted based on the procedure proposed by Yang et al. (2016) [23]. The used PCR primer pairs are shown in Table 1. These primers were commercially synthesized by Sangon Biotech Institute Co ltd., Shanghai, China.

### 4.8. Western Blot Analysis

Total protein was extracted using a Radio-Immunoprecipitation Assay (RIPA) lysis buffer. An equivalent of 15–30 µg protein extract was dissolved using sodium dodecyl sulfate–polyacrylamide gel electrophoresis and transferred on to a nitrocellulose membrane, which was blocked with Bovine Serum Albumin (BSA) for 2 h. Then, the membrane was probed with primary antibodies against Nrf2, GSH-Px, HO-1, γ-GCS, NQO1, and β-actin overnight at 4 °C and a secondary antibody at room temperature (Santa Fe, NM, USA). The image analysis system (Super ECL Plus, Applygen, Beijing, China) was used for the quantitative analysis of target protein expression.

### 4.9. Statistical Analysis

The SPSS17.0 software was used for the standard deviations (IBM, Almon, NY, USA). The differences among the groups were analyzed using analysis of variance (discrete analysis), and the least significant difference was used for multiple comparisons. The results were expressed as mean ± standard deviation. A *p* value <0.05 indicated a difference, and a *p* value <0.01 indicated a significant difference.

## Figures and Tables

**Table 1:** Primer sequence.

Gene	Accession No.	Primer Sequence (5′–3′)	Product Length
*Nrf2*	NM_010902.3	Forward: TCCTATGCGTGAATCCCAAT	103 bp
		Reverse: GCGGCTTGAATGTTTGTCTT	
*GSH-PX*	X03920.1	Forward: GAAGTGCGAAGTGAATGG	224 bp
		Reverse: TGTCGATGGTACGAAAGC	
*HO-1*	NM_010442.2	Forward: GGGCTGTGAACTCTGTCCAAT	162 bp
		Reverse: GGTGAGGGAACTGTGTCAGG	
*γ-GCS*	U85414.1	Forward: TGGATGATGCCAACGAGTC	185 bp
		Reverse: CCTAGTGAGCAGTACCACGAATA	
*NQO1*	NM_008706.5	Forward: TTCTGTGGCTTCCAGGTCTT	104 bp
		Reverse: TCCAGACGTTTCTTCCATCC	
*β-actin*	BC138614.1	Forward: CTGTCCCTGTATGCCTCTG	221 bp
		Reverse: TTGATGTCACGCACGATT	
